# Closed Complete Genome Sequences of Two Nontypeable Haemophilus influenzae Strains Containing Novel *modA* Alleles from the Sputum of Patients with Chronic Obstructive Pulmonary Disease

**DOI:** 10.1128/MRA.00821-18

**Published:** 2018-07-19

**Authors:** John M. Atack, Timothy F. Murphy, Lauren O. Bakaletz, Kate L. Seib, Michael P. Jennings

**Affiliations:** a Institute for Glycomics, Griffith University, Gold Coast, Queensland, Australia; b Clinical and Translational Research Center, University at Buffalo, State University of New York, Buffalo, New York, USA; c Center for Microbial Pathogenesis, The Research Institute at Nationwide Children’s Hospital and The Ohio State University College of Medicine, Columbus, Ohio, USA; Indiana University Bloomington

## Abstract

Nontypeable Haemophilus influenzae (NTHi) is an important bacterial pathogen that causes otitis media and exacerbations of chronic obstructive pulmonary disease (COPD). Here, we report the complete genome sequences of NTHi strains 10P129H1 and 84P36H1, isolated from COPD patients, which contain the phase-variable epigenetic regulators ModA15 and ModA18, respectively.

## ANNOUNCEMENT

Nontypeable Haemophilus influenzae (NTHi) is responsible for human respiratory tract infections (1, 2). Previous work characterizing NTHi showed that phase-variable N^6^-adenine DNA methyltransferases (ModA) are involved in epigenetic regulation and virulence (3–6). Phase-variable methyltransferase expression leads to genome-wide methylation differences, epigenetically regulating multiple genes—a phase-variable regulon (phasevarion) (7, 8). *modA* alleles show high variability (<25% nucleotide identity) in their central target recognition domain (TRD), which dictates specificity (9). Different TRDs methylate different sequences and define a phasevarion (7). We have shown that ∼65% of otitis media (OM) clinical isolates possessed one of five *modA* alleles, *modA2*, -*4*, -*5*, -*9*, or -*10* (4). Examination of *modA* alleles present in NTHi from a clinical collection of sputum samples from COPD patients (10) revealed two uncharacterized *modA* alleles, *modA15* and *modA18*.

We picked two strains, each containing a new *modA* allele (strain 10P129H1 contains *modA15*; strain 84P36H1 contains *modA18*), for genome sequencing and methylome analysis. DNA was sequenced at the Yale Center for Genome Analysis (YCGA) using a PacBio RS II platform with P6-C4 chemistry and a library size of 10 kb, with one strain per single-molecule real-time (SMRT) cell, and assembled *de novo* using the Hierarchical Genome Assembly Process (HGAP) (11). Preassembly was carried out using Celera Assembler v8.1 to the unitig step followed by a custom unitig consensus caller (YCGA). The first set of alignments was found by querying an index of the reference genome and then refining until only high-scoring alignments were retained (YCGA). Polishing for a pure PacBio assembly was carried out using the Quiver algorithm. Consensus sequences were submitted to NCBI for annotation with the Prokaryotic Genome Annotation Pipeline (PGAP), and annotated sequences were submitted to GenBank.

**FIG 1:**
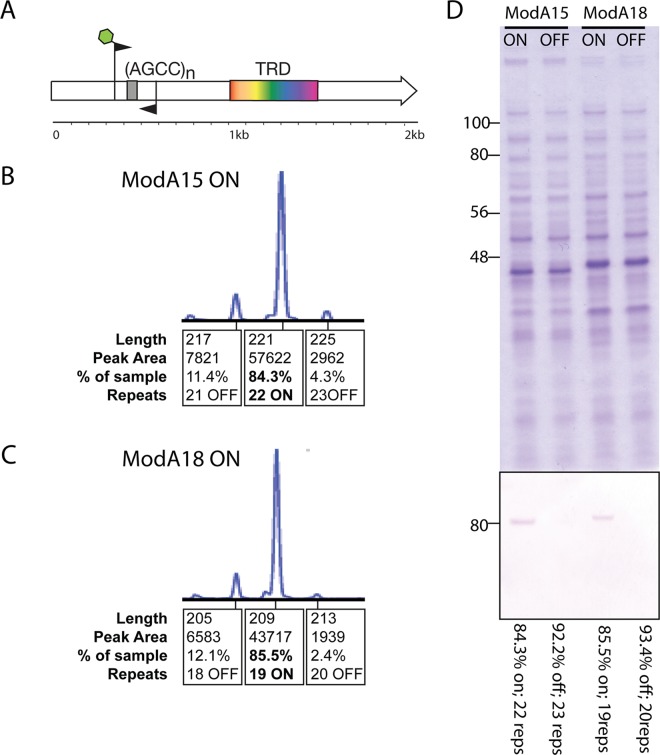
Confirmation of the on/off status of *modA15* and *modA18* in the studied strains. (A) *modA* gene, showing the location of the target recognition domain (TRD), which is highly variable between each *modA* allele and dictates the specificity of the ModA protein. The 5′ and 3′ regions are highly conserved (95% identity) between alleles. The location of the primers used for fragment analysis by performing PCR over the AGCC_(n)_ repeat tracks is shown, with the forward primer containing a fluorescent FAM (6-fluorescein) label (green hexagon) so fragments can be sized by GenScanner. (B and C) Fragment analysis traces of *modA15* on and *modA18* on strains showing that the majority of the bacterial population contains 22 AGCC repeats (*modA15*) and 19 AGCC repeats (*modA18*) in their open reading frame, meaning the gene is in frame, i.e., is on and therefore expressed. (D) Western blot and accompanying Coomassie stain of the *modA15* and *modA18* on strains with paired isolates of the same strain where the *modA* gene is out of frame, i.e., off, and not expressed.

NTHi strain 10P129H1 resolved into a genome of 2,047,595 bp with a G+C content of 37.9% and containing 2,079 open reading frames (ORFs). Strain 10P129H1 encodes *modA15*, containing 5′-AGCC_(16)_ repeats in its ORF. NTHi strain 84P36H1 resolved into a genome of 2,025,527 bp with a G+C content of 38.4% and containing 2,115 ORFs. Strain 84P36H1 encodes the *modA18* allele, containing 5′-AGCC_(19)_ repeats in its ORF. *modA* in both strains is expressed (on) with the strains’ respective numbers of AGCC_(n)_ repeats. The pregenome on/off status of *modA15* and *modA18* was verified using our fragment analysis approach as detailed previously (4) (Fig. 1). We also used Western blotting using an anti-ModA antibody to verify the presence of ModA in the culture used for DNA preparation for SMRT sequencing. Western blotting was carried out as described previously (4) (Fig. 1).

Strain 84P36H1 contains major NTHi virulence factors, such as lipooligosaccharide biosynthetic loci, and genes encoding the adhesins HMW1 and HMW2. Strain 10P129H1 contains a number of features associated with Haemophilus influenzae biogroup aegyptius (12), including a number of autotransporter adhesins and biogroup aegyptius-specific high-molecular-weight (HMW) proteins containing an octanucleotide 5′-GCATCATC_(n)_ repeat in their upstream region (12).

These data provide insight into the pathobiology of NTHi and will aid in the development of novel vaccines and antibacterial strategies.

### Data availability.

The complete genome sequences of the Haemophilus influenzae strains described in this article have been deposited in NCBI GenBank under the accession numbers CP029620 (10P129H1) and CP029621 (84P36H1).
